# Pathogenic Role of Complement in Antiphospholipid Syndrome and Therapeutic Implications

**DOI:** 10.3389/fimmu.2018.01388

**Published:** 2018-06-19

**Authors:** Francesco Tedesco, Maria Orietta Borghi, Maria Gerosa, Cecilia Beatrice Chighizola, Paolo Macor, Paola Adele Lonati, Alessandro Gulino, Beatrice Belmonte, Pier Luigi Meroni

**Affiliations:** ^1^Immunorheumatology Research Laboratory, Istituto Auxologico Italiano, Milan, Italy; ^2^Department of Clinical Sciences and Community Health, University of Milan, Milan, Italy; ^3^Department of Life Sciences, University of Trieste, Trieste, Italy; ^4^Tumor Immunology Unit, Human Pathology Section, Department of Health Sciences, University of Palermo, Palermo, Italy

**Keywords:** complement, antiphospholipid syndrome, anti-beta2 glycoprotein I antibodies, thrombosis, miscarriages, animal models, inflammation, therapy

## Abstract

Antiphospholipid syndrome (APS) is an acquired autoimmune disease characterized by thromboembolic events, pregnancy morbidity, and the presence of antiphospholipid (aPL) antibodies. There is sound evidence that aPL act as pathogenic autoantibodies being responsible for vascular clots and miscarriages. However, the exact mechanisms involved in the clinical manifestations of the syndrome are still a matter of investigation. In particular, while vascular thrombosis is apparently not associated with inflammation, the pathogenesis of miscarriages can be explained only in part by the aPL-mediated hypercoagulable state and additional non-thrombotic effects, including placental inflammation, have been described. Despite this difference, evidence obtained from animal models and studies in APS patients support the conclusion that complement activation is a common denominator in both vascular and obstetric APS. Tissue-bound aPL rather than circulating aPL-beta2 glycoprotein I immune complexes seem to be responsible for the activation of the classical and the alternative complement pathways. The critical role of complement is supported by the finding that complement-deficient animals are protected from the pathogenic effect of passively infused aPL and similar results have been obtained blocking complement activation. Moreover, elevated levels of complement activation products in the absence of abnormalities in regulatory molecules have been found in the plasma of APS patients, strongly suggesting that the activation of complement cascade is the result of aPL binding to the target antigen rather than of a defective regulation. Placental complement deposits represent a further marker of complement activation both in animals and in patients, and there is also some suggestive evidence that complement activation products are deposited in the affected vessels. The aim of this review is to analyze the state of the art of complement involvement in the pathogenesis of APS in order to provide insights into the role of this system as predictive biomarker for the clinical manifestations and as therapeutic target.

## Introduction

In recent years, major efforts have been made to define the molecular mechanisms responsible for the clinical manifestations of antiphospholipid syndrome (APS) including vascular thrombosis and adverse pregnancy outcomes (1–3). Blood clots can occur in both venous and arterial vessels with preferential localization in the brain and coronary arteries, although other vascular districts can also be involved. Vascular thrombosis mediated by beta2 glycoprotein I (β2GPI)-dependent antiphospholipid antibodies (aPL) represents the main pathogenic mechanism that is responsible for the major clinical manifestations of the syndrome and it has been suggested to be the cause also for other non-classification clinical events (4).

Pregnancy morbidity manifests as unexplained deaths of a morphologically normal fetus at or beyond the 10th week of gestation, eclampsia, or severe preeclampsia, particularly early, severe preeclampsia (5). Although it is clear that the specific antigenic reactivity of aPL and their targeting to the placenta are critical to produce their effect, pathogenic mechanisms that damage the fetal–maternal unit and cause abnormal placental development are incompletely understood (6). Indeed, the *in vivo* antigenic targets of lupus anticoagulant (LA), the strongest risk factor for adverse pregnancy outcomes in APS patients, are not known (7). While blood clotting represents the main clinical manifestation of vascular APS, non-thrombotic mechanisms have been suggested to be a more important cause of defective placentation characteristic of the syndrome (1). Moreover, although most patients display both manifestations, isolated vascular or obstetric variants can also be found and there is some discussion as to whether vascular and obstetric APS are the same disease (8).

Despite the fact that not all the animal models of aPL-mediated fetal loss display inflammatory signature at the placental level, inflammation has been suggested to play a role in APS miscarriages (9). Analysis of human placental tissues has not clarified this issue, since an inflammatory infiltrate was reported in the decidua only in some but not all studies. No sign of inflammation was observed in the vessel wall of human tissues at variance with the findings in obstetric APS. However, endothelial perturbation with the expression of a pro-thrombotic and pro-inflammatory phenotype was reported in APS models (10).

Complement (C) activation has been shown to be critical in APS models, since its blockade protects animals from both aPL-mediated clotting and fetal loss (11). In line with the data obtained in animal models, C was suggested to be involved in vascular APS following the observation of increased plasma levels of activation products and reduced C3 and C4 levels or CH50 activity in some patients (12–18).

Similar findings were reported in obstetric APS and C deposits were detected at placental level in some but not all studies (19–21). Moreover, the beneficial effect of eculizumab, a human monoclonal antibody that prevents C activation by neutralizing C5, observed in individual cases further supports the role of C activation in human APS (22).

## Complement and Vascular APS

Anecdotal reports revealed the involvement of C in vascular APS many years before the sound evidence of C contribution to both fetal loss and thrombosis models. Low serum levels of C4 were reported in a patient with thrombosis, miscarriages, and aPL, and C4 null alleles and low C3/C4 were found to be associated with aPL in systemic lupus erythematosus (SLE) patients (12, 14). Moreover, the suggestion that C is involved in APS was strongly supported by the demonstration of increased plasma levels of soluble C5b-9 in nearly 40% of a small series of APS patients with stroke (13).

Despite these early reports, no further studies on C activation in vascular APS have been carried out for a long time. Oku et al. reported reduced serum levels of C3, C4, and decreased CH50 activity in a small series of primary APS (PAPS). C consumption was associated with increased levels of the activation products C3a, C4a but not C5a in the absence of reduction of regulatory proteins factor H and I suggesting an enhanced turnover rather than a defective C regulation (15). However, no clear relationship was found between this finding and clinical or serological APS parameters (15). Similar increase in plasma levels of the C activation products Bb and C3a were reported by other groups in large series of patients with both vascular and obstetric PAPS (16, 18). The plasma levels of C3a were also found to be higher in another small series of persistently LA positive patients with no correlation with thrombosis (17). Moreover, we recently observed a significant increase in platelet- and red blood cell-bound C4d in PAPS in comparison to SLE and healthy controls further supporting the occurrence of C activation in the syndrome (23). C consumption and release of C activation products have been reported in a case of catastrophic APS (CAPS) (24).

The mechanism responsible for C activation has not been clarified. In one study, circulating immune complexes (CIC) found in a high proportion of PAPS patients have been suggested to trigger the classical pathway (15). The prevalence of CIC in APS was much lower in other studies suggesting that additional mechanisms may be responsible for C activation (16, 25). However, the issue is still a matter of research due to technical problems in detecting CIC.

C activation in the fluid phase can be associated with C deposition at tissue level, but few reports have been published documenting localization of tissue-bound C in APS patients. Immunoglobulin (Ig), C1q, and C3 deposits were described in the heart valve leaflets from patients with aPL-related valvulopathy (26), and Ig, C1q, and C3 have also been found in kidney biopsies from some but not all patients with APS nephropathy (27). Altogether, these findings suggest that aPL-mediated C activation can take place in the tissues of patients affected by this syndrome. We recently reported the case of a PAPS patient with arterial popliteal thrombosis who underwent arterial surgical bypass. Deposits of C1q, C4, C3, and C5b-9 co-localizing with β2GPI and IgG were found in the affected artery wall together with increased plasma levels of C5a and C5b-9. Interestingly, a short treatment with eculizumab resulted in a substantial decrease in the C5a and C5b-9 levels. Overall, these findings strongly suggest that C activation takes place in vascular APS and that C deposition at the anatomical site of thrombosis plays a key role in aPL-mediated clotting (28).

## The Role of Complement in Models of Vascular APS

Animal models of vascular APS have been instrumental in establishing the pathogenic role of antibodies to β2GPI in the formation of thrombi and the mechanism of their action. The model has been reproduced in various animal species including mice, hamsters, and rats using different experimental approaches.

APL-treated mice have been used extensively to monitor the development of thrombi in the femoral vein after a pinch injury (10). A somewhat similar approach was adopted in hamsters and mice that received monoclonal or patients’ antibodies to β2GPI, respectively, to induce clot formation in the carotid artery (hamster) or cremaster muscle microcirculation (mice) injured either by a photochemical reaction (29) or following laser exposure (30). Both approaches rely on mechanical or chemical vascular damage to initiate the coagulation process that is further enhanced by the administration of aPL resulting in enlargement of the blood clot. We followed a different strategy establishing a rat model that, in our view, reflects more closely the situation in the clinic (31). The model consisted of priming the animals with an amount of LPS, that does not induce thrombosis, followed by administration of aPL. Formation of thrombi in the mesenteric microvessels containing arterioles, capillaries, and postcapillary venules was monitored by optical imaging. This approach allowed us to firmly establish that aPL was totally ineffective in naïve animals and that the pro-coagulant effect of these antibodies required a second hit provided by LPS that was not needed for their proabortive activity.

Despite the different experimental approaches in the mouse and rat models of vascular thrombosis, blockade of C activation with a neutralizing antibody to C5 was shown to prevent thrombus enlargement in mice (32) or clot formation in rats (31) suggesting the important contribution of C to aPL-induced promotion of coagulation. The finding that aPL fail to exert a pro-coagulant effect in C3- and C5-deficient mice is consistent with the conclusion that C plays a critical role in mediating the damaging effect of the antibodies (32). However, there is a major difference in the mechanisms of C-mediated pregnancy loss and vascular thrombosis. While C5a has been shown to play a major role in causing adverse pregnancy outcome induced by aPL (33), blood clot formation is apparently dependent on the action of the terminal complex C5b-9, as suggested by the failure of aPL to promote thrombosis in C6-deficient rats and mice (31, 34). Deposition of C9 at sites of localization of IgG on the endothelium of the mesenteric microvessels of rats treated with aPL is a clear indication that C activation proceeds till the assembly of the membrane attack complex. We and others have provided evidence that the terminal complex either in a sublytic or cytolytically inactive form can stimulate endothelial cell to express on their surface tissue factor that triggers the extrinsic pathway of coagulation (35, 36).

## Complement and Obstetric APS

Studies in humans support the role of C in aPL-associated pregnancy complications. Mild hypocomplementemia and low C3, C4 levels were reported in some studies including aPL-positive patients with no other associated systemic autoimmune diseases (16, 37–39). Although this finding is suggestive for C involvement in aPL-mediated miscarriages, C activity was not reduced in all pregnant women and a clear relationship with pregnancy complications was not supported by statistical analysis. Interpretation of C levels in pregnant women is difficult because they reflect both increased synthesis stimulated by estrogens and consumption (40). To obtain a correct information on the C levels in aPL-positive pregnant women, the data should be compared with those found in normal pregnant controls, but this comparison was made only in one study (38).

More recently, increased plasma levels of the activation products Bb and C5b-9 were reported in women with aPL and adverse pregnancy outcome suggesting the contribution of C activated through the alternative pathway to the pathogenesis of this clinical condition (41). The activation products are considered a more sensitive marker of C activation, and may contribute to promote leukocyte recruitment/activation and release of pro-inflammatory and anti-angiogenic mediators responsible for placental damage. Deposition of C4d and to some extent of C3d in term placentas was reported in aPL women in two studies further suggesting the contribution of C activation to placental impairment mediated by aPL (19, 21).

## Complement Deposition on Human Placentas from APS Patients

While the finding of C activation products in the circulation of patients with obstetric APS is suggestive of C involvement in the pathogenesis of adverse pregnancy outcome, there is no doubt that the detection of these products in placenta provides more direct evidence for C contribution to tissue damage. Search for C deposits should of course be restricted to placentas from patients with PAPS to avoid confounding results that may derive from the analysis of tissue from patients with secondary APS associated with C-mediated disorders such as SLE. C localization was investigated in term placentas from patients with aPL antibodies by Shamonki and colleagues (19), who focused their analysis on the deposits of C4d, C3b, and C5b-9 complex. They reported the presence of these C activation products in the cytoplasm of villous trophoblast and on extravillous trophoblast, but it is unclear whether there was a preferential cytoplasmic localization also in these cells. Histologic examination of placentas revealed pathological lesions including decidual vasculopathy, increased syncytial knots, and villous infarcts, that were correlated with increased C4d staining of villous trophoblast. Surprisingly, the presence of C5b-9 in the cytoplasm of villous trophoblast was significantly lower suggesting that this complex may not contribute to tissue damage. In addition, the degree of C3b and C5b-9 deposition in extravillous trophoblast of placentas from aPL-positive patients was not significantly different from that found in control placentas raising the question of the relevance of these observations to the pathogenic role of C in aPL-mediated alterations in maternal decidua.

Our group has conducted a prospective study on 13 pregnancies in 11 patients with PAPS, who were under treatment with low molecular weight heparin (100 IU/kg/day s.c.) and low dose aspirin (100 mg/day). The majority of these patients (10 out 13) had medium to high titers of anti-β2GPI and positive LA. The pregnancies resulted in eight live births at gestational ages ranging between 30 and 38 weeks, one abortion and four fetal loss after 10 weeks’ gestation (Table 1). The study was approved by the Istituto Auxologico Italiano Ethics Committee (22-07-2010) and patients gave their written informed consent.

**Table 1:** Clinical characteristics of the PAPS patients examined for placental C deposits.

Patients	Diagnosis	LA	aCL IgG/IgM	anti-β2GPI IgG/IgM	Outcome	Therapy
BAC 1	PAPS	Pos	High/high	High/high	Fetal loss < 10 weeks	LMWH/ASA
BAC 2	PAPS	Pos	High/high	High/high	Fetal loss > 10 weeks (twins)	LMWH/ASA
BAC 3	PAPS	Pos	High/high	High/high	Live baby 35 weeks	LMWH/ASA/ivIg/CS
BA	PAPS	Pos	High/high	High/high	Fetal loss > 10 weeks	LMWH/ASA
TD	PAPS	Neg	Med/low	Med/neg	Live baby 38 weeks	LMWH/ASA
SA	PAPS	Pos	nd	nd	Fetal loss > 10 weeks	ASA^a^
SE	PAPS	Pos	High/high	High/med	Live baby 30 weeks	LMWH/ASA
PA	PAPS	Pos	High/high	nd	Fetal loss > 10 weeks	None^b^
FO	PAPS	Neg	High/neg	High/neg	Live baby 38 weeks	LMWH/ASA
BO	PAPS	Pos	Neg/neg	Neg/neg	Live baby 35 weeks	LMWH/ASA
PU	PAPS	Pos	High/neg	High/med	Live baby 38 weeks	LMWH/ASA
AC	PAPS	Neg	High/neg	High/med	Live baby 33 weeks	LMWH/ASA
BL	PAPS	Pos	High/med	High/med	Live baby 31 weeks	LMWH/ASA

*PAPS, primary antiphospholipid syndrome (5); C, complement; LA, lupus anticoagulant; aCL, anti-cardiolipin antibodies; anti-β_2_GPI, anti-beta2 glycoprotein I antibodies; LMWH, low molecular weight heparin; ASA, aspirin; ivIg, intravenous immunoglobulins; CS, corticosteroids; nd, not detected*.

*^a^The patient was classified as aPL-positive asymptomatic carrier, and her first pregnancy was treated with ASA only*.

*^b^The patient was not treated with the standard therapy because the positivity for aPL was found after the abortion*.

**Figure 1:**
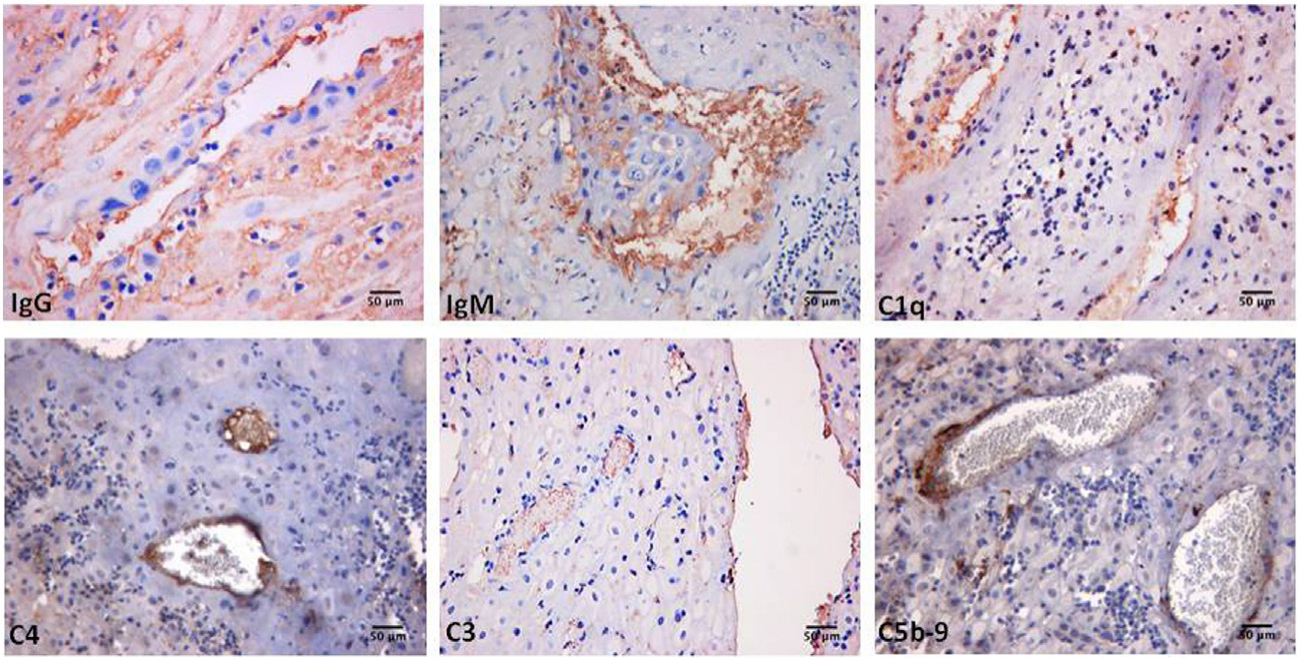
Immunoperoxidase staining of a representative placental decidua from a primary antiphospholipid syndrome patient showing deposition of immunoglobulin (Ig) and various C components (20× magnification).

**Figure 2:**
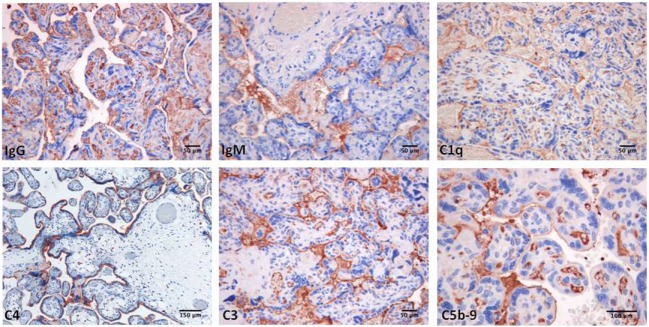
Immunoperoxidase staining of representative placental villi from a primary antiphospholipid syndrome patient showing deposition of immunoglobulin (Ig) and various C components (20× magnification).

Decidual vasculopathy and intervillous thrombi were the most common histologic findings observed in PAPS placentas while inflammation was less frequent and was seen in both PAPS and control placentas, as were also villitis and villous infarcts equally detected in both groups of placentas. Deposits of C components and C activation products were found in all PAPS placentas examined with some variation in the degree of C deposition. C1q, C4, and C3 were detected on the decidual endothelium vessels at sites of IgG and IgM deposition while C5b-9 showed a prevalent subendothelial distribution (Figure 1). Analysis of the villi revealed the presence of IgG, IgM, C1q, C4, C3, and C5b-9 on the surface of syncytiotrophoblast with additional distribution of IgM and C5b-9 on intervillous fibrin deposits and of IgG and C3 on the endothelium of villous vessels (Figure 2). Interestingly, C activation occurred in placental tissue of APS patients despite heparin treatment. This was a surprising finding because heparin was shown by Girardi and colleagues (42) to inhibit C activation and to prevent pregnancy loss in a mouse model of obstetric APS. C localization in decidua and villi of control placentas was negligible with the only exception of C1q detected on decidual endothelium and on extravillous trophoblast confirming previous observation that C1q is constitutively expressed in these cells in physiological pregnancy (43–45). Altogether, these findings support the conclusion that C activation in APS placenta is activated by aPL antibodies and justify a possible pathogenic role of C activation in fetal loss documented in this study and in murine models of APS. However, the presence of C deposits in placentas from patients who have had live births is more difficult to interpret. It is possible that the damaging effect of C activation on placental tissue that affects pregnancy outcome depends on the extent and distribution of C deposits in APS placenta. Binding of C activation products to restricted placental areas may cause tissue alterations that marginally affect the regular progression of pregnancy.

## The Role of Complement in Animal Models of Pregnancy

The initial observation by Branch and colleagues (46) that passive infusion of serum IgG from patients with aPL induced an increased rate of fetal resorptions in pregnant mice was the first evidence that suggested a role of the antibodies in the pathogenesis of fetal loss. These findings and similar data obtained by other groups (47, 48) led to the conclusion that obstetric APS is a clinical disorder mediated by antibodies that are preferentially directed against β2GPI (4). The high degree of protein sequence homology between human and animal β2GPI explains the ability of human antibodies to cause fetal demise in mice. The β2GPI molecule has been found to be localized in the placenta of pregnant mice in the absence of antibodies with a prevalent distribution on syncytio and extravillous trophoblasts, and decidual endothelial cells (49). *In vitro* experiments have shown that aPL interacting with the target molecule expressed on trophoblast impair several functions of these cells including proliferation, syncitia formation, invasion into maternal deciduas, as well as secretion of chorionic gonadotrophin and growth factors (1, 50). However, the *in vivo* relevance of these observations to placental dysfunction is unclear as the administration of aPL to C3-deficient pregnant mice has no adverse impact on the progression of pregnancy while resulting in an increased rate of fetal loss and growth retardation in wild-type animals (51). These data argue for a major role of C activated by aPL in inducing adverse pregnancy outcome, a conclusion which is also supported by the ability of the C3 convertase inhibitor Crry to prevent aPL-mediated fetal loss (51).

Further analysis of the critical step of the C sequence involved in this pathological process points to C5 as the key component based on the finding that aPL failed to increase fetal resorption rate in C5-deficient mice and in animals treated with anti-C5 antibodies (33). Similar results were obtained in C4 and factor B-deficient mice suggesting that C activation is triggered by aPL through the classical pathway and is further amplified through the alternative pathway (52). As activation of C5 results in the release of the small pro-inflammatory peptide C5a and the large fragment C5b that initiates the assembly of the terminal complex C5b-9, experiments conducted to clarify their relative contribution to fetal damage have led to the identification of C5a as the main mediator of fetal injury. The effect of C5a has been attributed to its ability to interact with C5aR expressed on PMN and to stimulate the release of TNF-α that induces apoptosis of cytotrophoblasts and promotes inflammation (53, 54). C5a was also found to induce expression of tissue factor in PMN that contributes to favor decidual inflammation and in turn increased fetal loss (55). The terminal complex does not seem to play a role in aPL-mediated fetal injury as C6-deficient mice had a similar rate of pregnancy loss as wild-type mice.

## Therapeutic Perspectives

A wealth of experimental and clinical data have clearly shown that C is implicated in the pathogenesis of the clinical manifestations of APS and have led to the suggestion that this syndrome should be considered a C-dependent disorder (31, 54). Despite the large body of evidence mainly obtained from animal models supporting this conclusion, antibodies or other reagents neutralizing C have not been regarded as first-line treatment to prevent adverse pregnancy outcome or thrombus formation. One possible explanation is that anticoagulants and low-dose aspirin have been used successfully to prevent vascular thrombosis and pregnancy abnormalities and there is a general consensus on their use in the primary treatment of APS patients. However, it is worth mentioning that this type of therapy is not always effective and does not always prevent recurrences of obstetric and vascular complications particularly in patients with triple positivity for LA, anti-cardiolipin, and anti-β2GPI antibodies (56). There are some suggestions that the overall therapeutic efficacy of anticoagulants can be increased by a combined treatment with other drugs, although specific clinical trials are still lacking (22).

Several case reports have been published describing patients with CAPS who have benefited from eculizumab administration with a substantial amelioration of their clinical manifestations and successful kidney transplantations have also been published (22, 57). Similar beneficial results were obtained blocking C activation in APS patients with multiple arterial thrombosis refractory to standard therapy (58). It must be pointed out that chronic administration of eculizumab would be an expensive therapy to prevent thrombus formation due to the high cost of treatment to avoid an unpredictable event. This therapeutic measure can satisfactorily be restricted to situations in which blood clots are more likely to occur. We have shown that eculizumab administered to an APS patient prior to femoro-popliteal bypass surgery aimed at removing arterial occlusion was effective in preventing re-thrombosis (28). As surgical intervention represents the second hit required for thrombus formation in APS patients with circulating aPL, eculizumab would be expected to have a beneficial effect in patients undergoing vascular surgery. One clinical trial is ongoing to assess the efficacy of eculizumab in the prevention of APS-associated thrombotic microangiopathy following renal transplantation (Clinical Trial.gov #: NCT01029587).

Pregnancy is more frequently susceptible to complications in APS patients as this condition provides by itself a second hit in addition to anti-β2GPI antibodies. Fortunately, a large proportion of APS pregnant women responds to the combination of heparin and low dose aspirin, but approximately 20% are resistant to the standard therapy (22). These patients and in particular those with a history of recurrent abortions may preferentially respond to treatment with C5-neutralizing antibody. Recent data have excluded any adverse effect of eculizumab on fetus as only a negligible amount of antibody administered to pregnant women crosses the placenta leaving the C activity of the newborn largely unaffected (58, 59). We propose an alternative option based on the use of a human anti-β2GPI monoclonal antibody that targets the antigen highly expressed on syncytiotrophast, extravillous trophoblast, and decidual endothelial cells and is functionally ineffective being unable to activate C (60).

## Conclusion

Experimental and clinical data accumulated over recent years support the conclusion that the C system is a key factor in the pathogenesis of the clinical manifestations of both vascular and obstetric APS. In fact, all the *in vivo* models and the reports from human studies clearly showed C activation; on the other hand, *in vitro* experimental models demonstrated that aPL may directly affect tissue targets or coagulation factors. Further studies are warranted to establish if C activation products should be considered useful markers to monitor disease severity and also if C neutralization should be included as first-line therapeutic option in APS patients with CAPS or scheduled for organ transplant or different vascular surgical procedures.

## Author Contributions

FT and PLM contributed to conception of the work and wrote the first draft of the manuscript. AG and BB provided the immunohistochemistry pictures. All authors contributed to manuscript revision, read and approved the submitted version.

## Conflict of Interest Statement

The authors declare that the research was conducted in the absence of any commercial or financial relationships that could be construed as a potential conflict of interest.
